# An m7G-related lncRNA signature predicts prognosis and reveals the immune microenvironment in bladder cancer

**DOI:** 10.1038/s41598-023-31424-y

**Published:** 2023-03-15

**Authors:** Zhenchi Li, Jie Zhao, Xing Huang, Jiangping Wang

**Affiliations:** 1 Department of Urology, The Affiliated Taizhou People’s Hospital of Nanjing Medical University, Taizhou School of Clinical Medicine, Nanjing Medical University, 366 Taihu Road, Taizhou, 225300 Jiangsu China; 2 Graduate School of Dalian Medical University, No. 9 West Section, Lushun South Road, Dalian, Liaoning China; 3 Department of General Surgery, Shengjing Hospital of China Medical University, Shenyang, Liaoning China

**Keywords:** Cancer genetics, Urological cancer

## Abstract

Bladder cancer (BC) is a representative malignant tumor type, and the significance of N7-methyguanosine (m7G)-related lncRNAs in BC is still unclear. Utilizing m7G-related lncRNAs, we developed a prognostic model to evaluate BC's prognosis and tumor immunity. First, we selected prognostic lncRNAs related to m7G by co-expression analysis and univariate Cox regression and identified two clusters by consensus clustering. The two clusters differed significantly in terms of overall survival, clinicopathological factors, and immune microenvironment. Then, we further constructed a linear stepwise regression signature by multivariate Cox and least absolute shrinkage and selection operator (LASSO) regression analysis. Patients fell into high-risk (HR) and low-risk (LR) groups considering the train group risk score. HR group had worse prognoses when stratified by clinicopathological factors. The receiver operating curve (ROC) suggested that the signature had a better prognostic value. Tumor mutation burden (TMB) showed a negative relevance to the risk score, and patients with low TMB presented a better prognosis. Validation of the signature was carried out with multivariate and univariate Cox regression analysis, nomogram, principal component analysis (PCA), C-Index, and quantitative reverse transcriptase PCR (qRT-PCR). Finally, the gene set enrichment analysis (GSEA) demonstrated the enrichment of tumor-related pathways in HR groups, and single-sample gene set enrichment analysis (ssGSEA) indicated a close association of risk score with tumor immunity. According to the drug sensitivity test, the signature could predict the effects of conventional chemotherapy drugs. In conclusion, our study indicates the close relevance of m7G-related lncRNAs to BC, and the established risk signature can effectively evaluate patient prognosis and tumor immunity and is expected to become a novel prognostic marker for BC patients.

## Introduction

Bladder cancer (BC) is a representative malignant tumor of the urinary system^1^. In about 75% of BC, non-muscle-invasive bladder cancer (NMIBC) has papillary morphology and low-grade malignancy. The rest 25% manifests as muscle-invasive bladder cancer (MIBC), a non-capillary high-grade tumor tending to spread regionally or systemically^2,3^. BC treatment options depend on the type of BC, the stage of the disease, and the extent to which it has metastasized^4^. Although most BC is NMIBC at first diagnosis, the risk of recurrence approaches 30%, and 10–30% of patients progress to MIBC^5^. When patients progress to the stage of muscle progression, the prognosis is usually poor, and patients with advanced metastatic BC are challenging to cure. Despite some improvements in anesthesia and surgery and the widespread use of perioperative chemotherapy, long-term patient survival has remained unchanged for decades^2^. Less than 1/3 of patients with metastatic BC have a durable response to chemotherapy, which is a significant clinical challenge^6,7^. Novel reliable signatures shall be identified to predict the BC prognosis, and new therapeutic targets shall be developed to improve the suboptimal prognosis.

Recently, RNA modification has been confirmed to be related to tumor pathophysiology, especially tumor immunity^8^. More than 170 RNA modifications recently have been discovered^9^, including 5-methylcytidine (m5C), N6-methyladenosine (m6A), and N7-methyguanosine (m7G)^10^. M7G, common post-transcriptional modifications of RNA, is essential for regulating gene expression. Increasing studies have implicated m7G in cancer development and occurrence, such as colon cancer, lung cancer, and hepatocellular carcinoma^11–15^. The M7G methylation complex includes METTL1 and WDR4^16^. Currently, a study revealed that METTL1-mediated m7G tRNA modification could enhance tumor cell proliferation, migration, and invasion by regulating EGFR/EFEMP1 translation^17^. In addition, RNA methylation modification has been shown to maintain T cell homeostasis. Loss of METTL3 precludes T cells from undergoing homeostatic expansion and arrests in the naive state segment for longer periods through METTL3-mediated m6A methylation targeting the IL-7/STAT5/SOCS pathway^18^. M6A methylation can maintain the ability of Regulatory T cells (Tregs) to inhibit T cell proliferation, which may hinder the tumor-killing function of CD8 + T cells^19^. There are relatively few reports on m7G methylation in tumor immunity.

Long non-coding RNA (lncRNA) refers to a type of RNA of which the length is over 200 nucleotides^20^. Despite no translational function, lncRNAs play essential roles in various physiological functions and biological processes^21^. Recently, it has been found that it participates in the occurrence and progression of tumors not only by changing the malignancy of cancer cells itself but also by changing the tumor immune microenvironment, and its abnormal expression can be used as an effective biomarker for early diagnosis and monitoring of treatment effects^22,23^. Initiation, progression, and treatment of BC seem to be influenced by lncRNAs^24^. LncRNA RP11-89 strengthened BC cell proliferation and migration^25^. LncRNA GAS6-AS2 is related to the BC stage and poor prognosis^26^. It has been found in some studies that m7G-associated lncRNAs are linked to tumor therapy and prognosis^27,28^, but their roles in the prognosis and treatment of BC are still unclear.

At present, tumor immunotherapy has made remarkable progress. Immune checkpoint inhibitors (ICIs) could help to treat advanced BC effectively. But the response rate of ICIs was 15–25%, with durable response only seen in some patients^1^. Therefore, with the emergence of drug resistance in patients, there is an increased need for biomarkers to predict treatment response and patient prognosis.

Sequencing data and clinical information about BC came from The Cancer Genome Atlas (TCGA). Then, prognostic m7G-related lncRNAs were recognized. Through cluster typing and constructing a prognostic signature of m7G-related lncRNA, we evaluated the correlation with immune and chemotherapeutic drugs and explored the relevance of risk score to BC.

## Methods

### Sample data collection

412 BC and 19 normal datasets came from the TCGA database (https://portal.gdc.cancer.gov/), including genomic, clinical, and somatic mutation data. By excluding duplicate data, data acquired by formalin-fixed paraffin-embedded tissues and data without a follow-up of more than 30 days, 390 BC and 19 normal datasets were finally included. The clinical data represented survival time, survival status, age, gender, tumor node metastasis classification, and stage (Table 1).

**Table 1:** Demographical characteristics and clinical data of the patients.

**Variables**	Patients (n = 390)
*Age (year)*	68 (60,76)
*Gender*	
Male	289 (74.1%)
Female	101 (25.9%)
*TNM stage*	
I	2 (0.5%)
II	122 (31.3%)
III	136 (34.9%)
IV	128 (32.8%)
Unknow	2 (0.5%)
*T*	
Tx	1 (0.3%)
T0	1 (0.3%)
T1	3 (0.8%)
T2	111 (28.5%)
T3	190 (48.7%)
T4	53 (13.6%)
Unknow	31 (7.9%)
*N*	
Nx	36 (9.2%)
N0	225 (57.7%)
N1	44 (11.3%)
N2	74 (19.0%)
N3	6 (1.5%)
Unknow	5 (1.3%)
*M*	
Mx	190 (48.7%)
M0	187 (47.9%)
M1	10 (2.6%)
Unknow	3 (0.8%)

### Identification of prognosis-related lncRNAs

Firstly, we retrieved 33 m7G-related genes from previous articles and the gene set enrichment analysis (GSEA) website (http://www.gsea-msigdb.org/). Then, we extracted 882 lncRNAs related to m7G using the “limma” package with |Pearson R|> 0.3 and *p* value < 0.001. In the Sankey diagram, lncRNAs and m7G-related genes are correlated in detail. Finally, we applied the "survival" package in the univariate Cox regression analysis (*P* < 0.01) to filter prognostic-related lncRNAs. The obtained m7G-related lncRNAs were displayed with a forest plot and heatmap.

### Consensus cluster analysis and immune analysis

By the “ConsensusClusterPlus” package, the samples were divided into 2 clusters by prognostic lncRNAs expression, and then the survival curves based on the “survival’ package and the “survminer” package served for comparing the survival differences between the subtypes. A heatmap showed the correlations between clusters and clinicopathological features to explore the clinical value of clusters, including age, gender, tumor node metastasis classification, and stage.

We investigated the relationship between clusters and immunity using the ESTIMATE algorithm to calculate each cluster's StromalScore, ImmuneScore, and ESTIMATEScore. By single-sample gene set enrichment analysis (ssGSEA) with the "GSVA" package in R software, we calculated the infiltration scores regarding 16 immune cells and immune-related pathways^29^. We compared immune checkpoints between the two clusters utilizing the packages “limma”, “reshape2”, “ggplot2”, and “ggpubr”.

We performed the LASSO Cox regression to determine the prognostic features of m7G-related lncRNAs^30^. We randomly divided all tumor samples into train and validation cohorts using a 1:1 ratio. The risk score calculation for every patient was based on a linear combination of signature-related lncRNAs expression value multiplied by its multivariate Cox regression coefficients. Therefore, Risk Score = Expression_lncRNA1_ × Coefficient_lncRNA1_ + Expression_lncRNA2_ × Coefficient_lncRNA2_ + ⋯ + Expression_lncRNAn_ × Coefficient_lncRNAn_. Afterward, we took the median risk score as the cut-off point for dividing patients into HR and LR groups.

Survival analysis was conducted on the two groups, and the “timeROC” package served for the generation of the receiver operating curve (ROC) and area under the curve (AUC) for testing the prediction signature's accuracy. Univariate and multivariate Cox regression assisted in assessing the independent prognostic significance of risk score and clinicopathological characteristics.

### Nomogram and principal component analysis (PCA)

For the evaluation of the overall survival (OS) for each patient, the risk score and clinical characteristics (age, TNM stage, and stage) were taken into account to construct a prognostic nomogram, using the “rms” package to assess the 1-, 3-, and 5- year survival probability. We formulated the calibration curves to evaluate the consistency between the predictive and actual survival rates and calculated the consistency index (C-index). PCA was created to examine patient distribution in different groups by the “scatterplot3D” R package.

### Bioinformatics analysis

We assessed the proportion of immune-infiltrating cells between HR and LR groups according to the TIMER, CIBERSORT, CIBERSORT–ABS, QUANTISEQ, MCPCOUNTER, XCELL, and TIMER algorithms^31–36^. GSEA software (version 4.3.2, http://www.broad.mit.edu/gsea/) was utilized to detect possible functional pathways^37–39^. ssGSEA was conducted on the HR and LR cohorts by the “GSVA” package to compute the immune cell infiltration score and the immune function and further compare the difference between the two groups in terms of the expression of immune checkpoints^40^. An analysis of the somatic mutation between groups was performed utilizing a “maftools” package according to the established risk signature. Then, the “ggpubr” package was used to visualize tumor mutational burden (TMB). Survival curves were visualized utilizing the “survival” and “survminer” packages, and the association between risk classification and mutation classification was explored. Finally, to determine how HR and LR groups respond differently to chemotherapeutic drugs, we utilized the “pRRophetic” package to predict the half-maximal inhibitory concentration (IC50) for different chemotherapeutic drugs.

### RNA isolation and quantitative reverse transcriptase PCR (qRT-PCR)

Normal bladder epithelial cells (SV-HUC-1) and BC cells (5637, T24) were provided by the Cell Bank of the Shanghai Institute of Cell Research. All lncRNAs were obtained using FastPure Cell/Tissue Total RNA Isolation Kit V2 (cat# RC112-01; Vazyme, China). Next, the HiScript® II Q RT SuperMix for qPCR (+gDNA wiper) (cat# R223; Vazyme) was used to reverse the extracted lncRNA for obtaining cDNA. Subsequently, RT-qPCR was finished using ChamQ SYBR qPCR Master Mix (cat# Q311-02; Vazyme). Thermal cycling conditions for RT-qPCR: 95 °C, 180 s; 95 °C, 10 s; 62 °C, 40 s; 40 cycles. Table S1 lists the primer sequences.

### Statistical analysis

R software (https://www.r-project.org/, R version 4.2.1) served for all the statistical analyses. The prognostic value of the risk signature was evaluated by Cox regression, and correlations between variables were tested utilizing Pearson's correlation test. OS was analyzed utilizing Kaplan–Meier (KM) and log-rank tests. The Student's test served for comparing data with normal distribution, the Mann–Whitney U test for comparing data with abnormal distribution, and the chi-square test for comparing categorization variables.

## Results

### Prognosis-related lncRNAs with coexpression of m7G in BC

**Figure 1:**
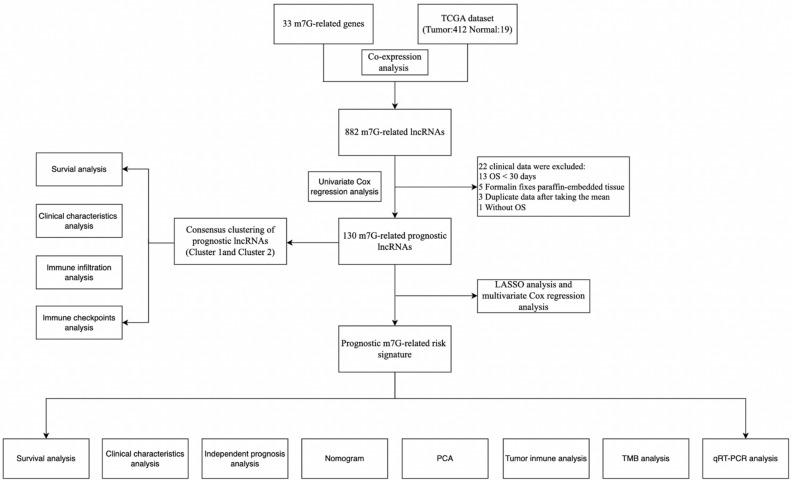
The flow diagram.

**Figure 2:**
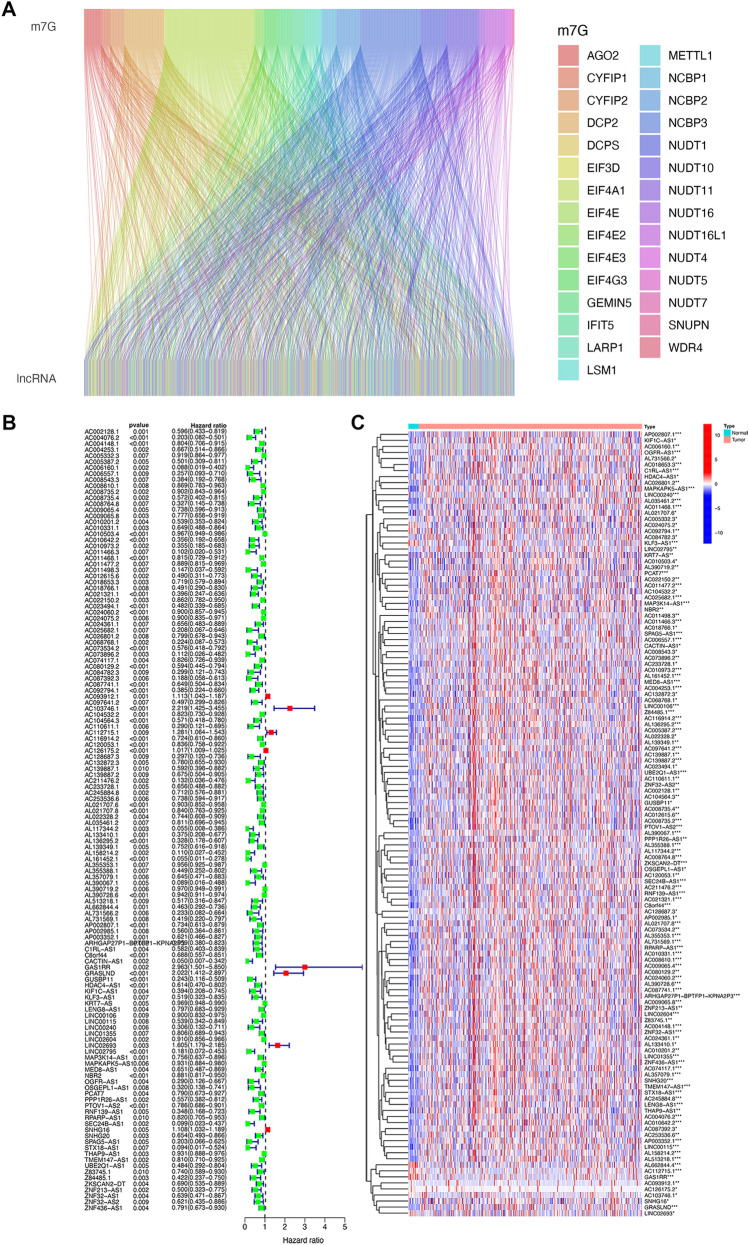
Identification of m7G-ralated prognostic lncRNAs. (**A**) Sankey diagram showing that correlation between m7G-related genes and lncRNAs. (**B**) Forest plot of the univariate Cox regression analysis between the 130 m7G-related lncRNAs and overall survival (OS) in BC patients. (**C**) Heatmap of differently expressed m7G-related lncRNAs between tumor and normal issues. The heatmap was created by using “pheatmap” package in R 4.2.1 (https://www.r-project.org/). *P < 0.05; **P < 0.01; ***P < 0.001.

The overall process is shown in Fig. 1. We extracted 17,876 lncRNAs from the TCGA database with expression data. The Sankey diagram visualized 882 lncRNA co-expression relationship by Pearson's correlation analysis according to the identified m7G-related genes (Fig. 2A). On this basis, 130 lncRNAs were related to BC prognosis using univariate Cox regression (Fig. 2B,C).

### Consensus clustering of m7G-related prognostic lncRNAs

**Figure 3:**
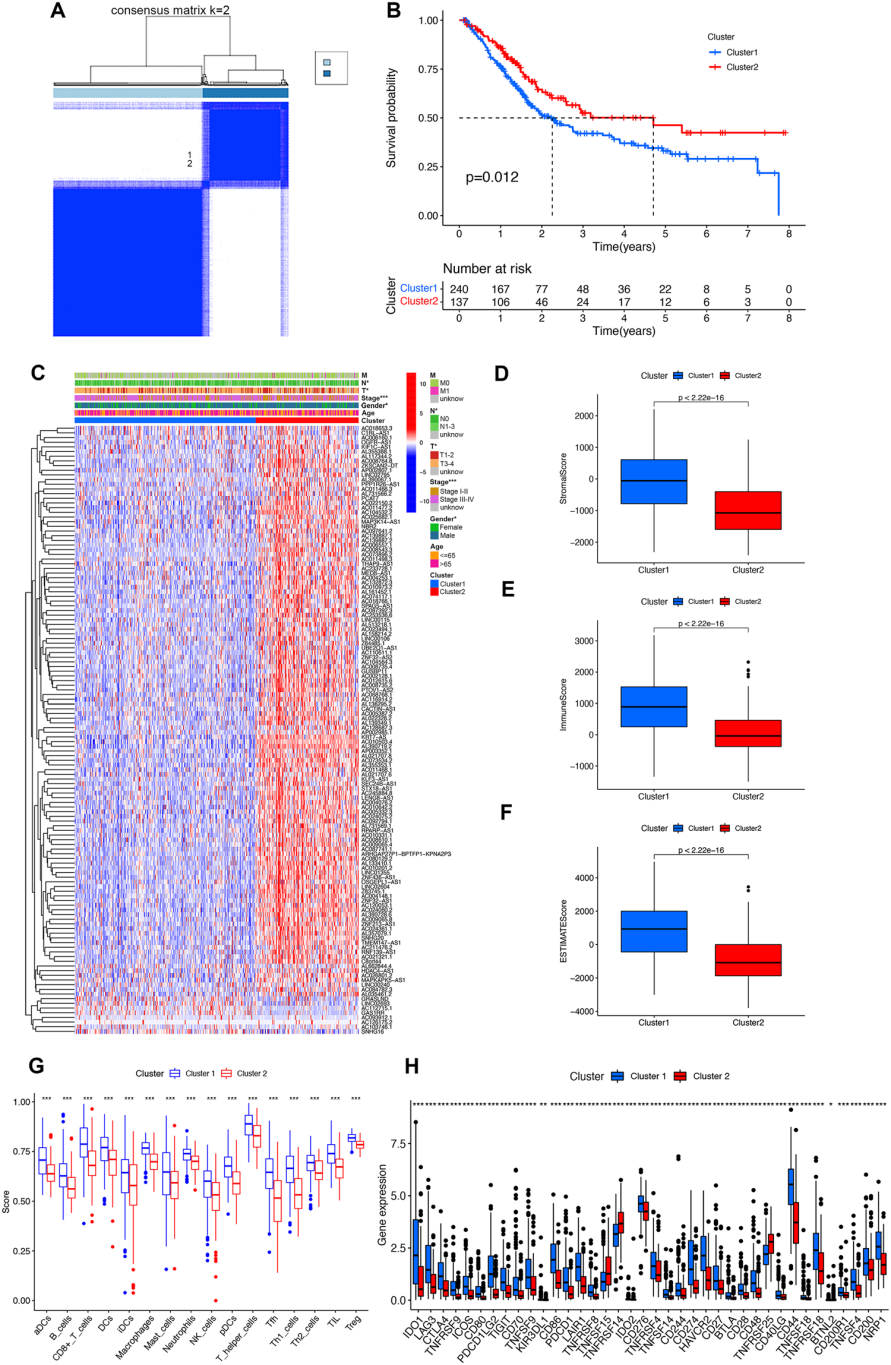
Consensus clustering analysis of the m7G prognostic lncRNAs and tumor immune cell microenvironments in cluster 1 and cluster 2. (**A**) Consensus clustering cumulative distribution feature for k = 2, (**B**) Kaplan–Meier curves for the OS of patients in two clusters. (**C**) Heatmap of the two clusters along with clinicopathological characteristics. The heatmap was created by using “pheatmap” package in R 4.2.1 (https://www.r-project.org/). (**D**–**F**) Patients with bladder cancer in cluster 1 had higher stromal score, immune score and ESTIMATE score compared to cluster 2. (**G**) Abundance of 16 infiltrating immune cell types in two clusters. (**H**) The expression levels of immune checkpoint in cluster 1 and cluster 2. *P < 0.05; **P < 0.01; ***P < 0.001.

Prognostic-related lncRNAs were clustered to assess the significance of m7G-related prognostic lncRNAs in developing BC. The best clustering was displayed when the cluster variable was set to 2 (Fig. 3A, Fig. S1A–C). The KM curves revealed that cluster 2's OS rate was higher than cluster 1 (Fig. 3B). Also, most m7G-related lncRNAs' expression level was lower in cluster 1 than in cluster 2 (Fig. 3C). Clinicopathological factors and clusters differed significantly. Compared to cluster 1, cluster 2, and T1-2, N0, stage I-II, and males were correlated (Fig. 3C). Therefore, the clustering revealed that the expression patterns of m7G-related lncRNAs were strongly associated with the malignancy of BC.

### Correlation between consensus cluster and immune infiltration

From an immunological perspective, we explored clusters' role in tumor microenvironments. Cluster 1 had higher StromalScores, ImmuneScores, and ESTIMATEScores than cluster 2, and its tumor purity was lower (Fig. 3D–F). Meanwhile, ssGSEA revealed the majority of cell subtypes (B cells, CD8 + T cells, and T regulatory cells (Tregs)) were enriched in cluster 1 (Fig. 3G). In addition, more immune checkpoint inhibitory molecules in cluster 1 were highly expressed (PDCD1 (PD-1) and CD274 (PD-L1), as well as CTLA4 (Fig. 3H).

### Establishment of a prognostic risk signature

**Figure 4:**
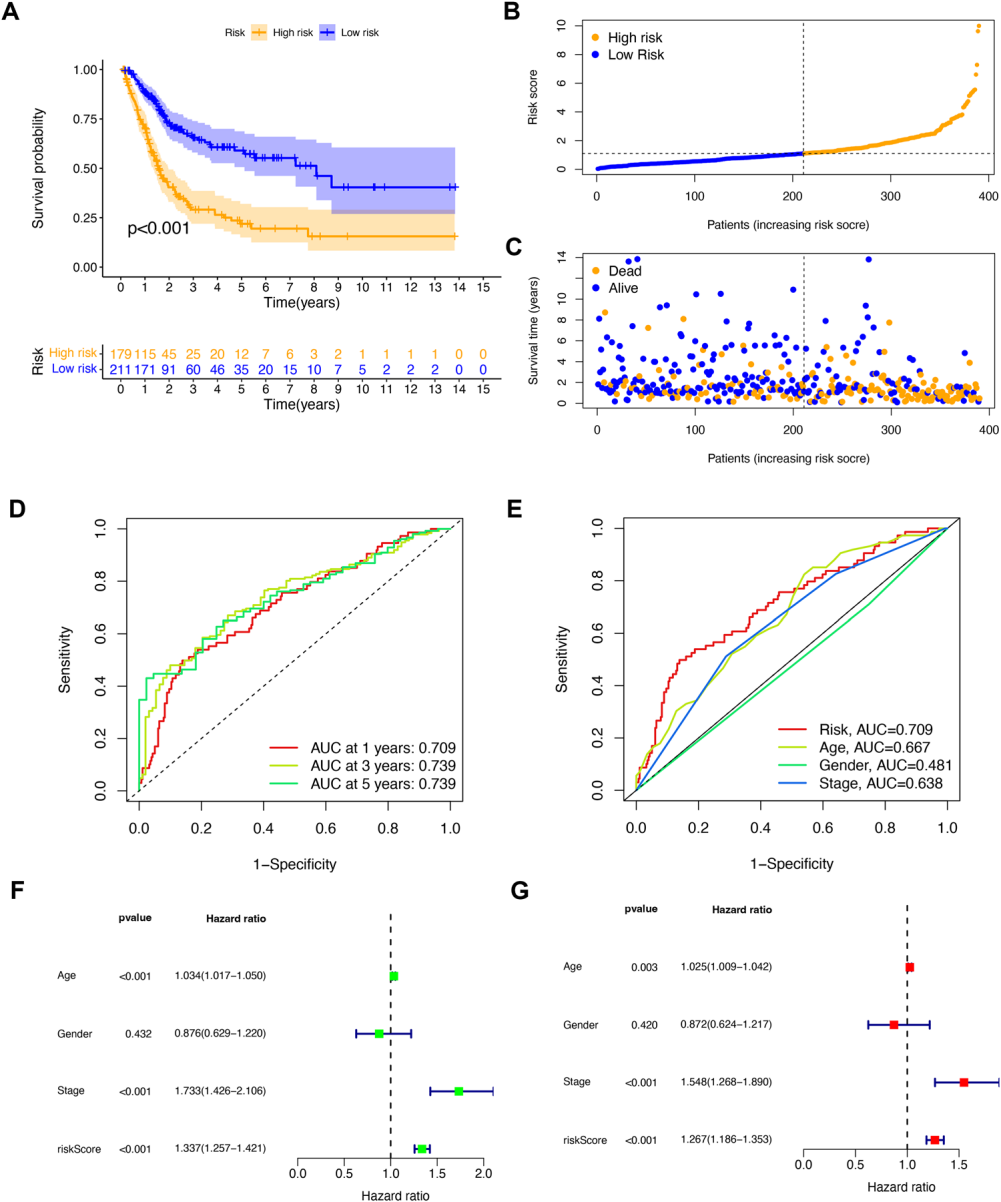
Establishment of the risk signature on m7G-related prognostic lncRNAs. (**A**) Kaplan–Meier curves for the OS of patients in the high- and low-risk groups. (**B**) The distributions of risk scores in the risk signature. (**C**) The distributions of survival status with different risk scores. (**D**) Time-dependent receiver operating characteristic (ROC) curves for predicting 1-year, 3-year, and 5-year OS in the TCGA cohort. (**E**) The ROC curves of the risk score and clinicopathological features. (**F**) Forest plot showing the results of univariate Cox regression analysis for all tumor samples. (**G**) Forest plot showing the results of multiple Cox regression analysis for all tumor samples.

We filtered 130 prognosis-related lncRNAs utilizing univariate regression, identified 23 robust prognosis-related lncRNAs utilizing LASSO regression, and selected eight lncRNAs based on multivariate Cox regression to construct a BC risk score signature (Fig. S2A,B). The risk score was computed using the formula: Risk Score = (− 2.2218 * AC006160.1) + (− 0.4732 * AC018653.3) + (− 0.4662 * AL035461.2) + (− 0.4973 * AL662844.4) + (0.7526 * GRASLND) + (− 0.6210 * HDAC4-AS1) + (0.6509 * LINC02693) + (− 0.3925 * PCAT7). Based on KM analysis, patients in the HR group presented a poorer survival probability than the LR group (Fig. 4A).

Risk outcomes and survival status showed a significant increase in patient mortality with increasing risk scores (Fig. 4B,C). Time-dependent ROC curves determined the risk score of 1-, 3-, and 5-year prognosis, and the corresponding AUC values were 0.709, 0.739, and 0.739, respectively (Fig. 4D). Furthermore, the AUC value of clinicopathological factors was lower than that of the risk score (Fig. 4E). We performed univariate and multivariate Cox regression. We found that the m7G-related signature was an independent prognostic predictor for OS in BC (Fig. 4F,G).

### Internal validation of predictive signature confidence

**Figure 5:**
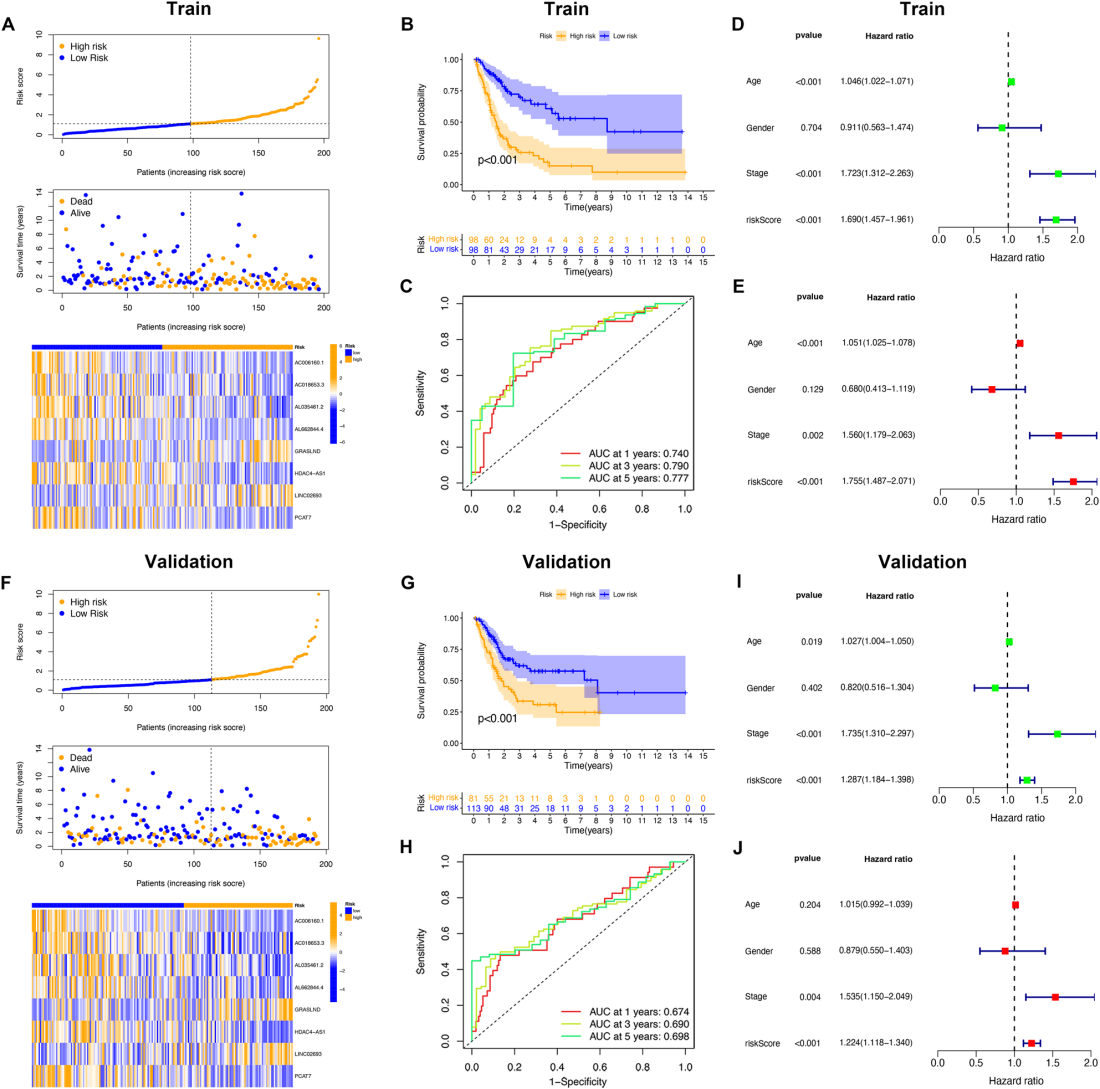
Internal validation of the risk signature in the TCGA database. (**A**) The distributions of risk scores and survival status in the train cohort. (**B**) Kaplan–Meier curves for the OS of patients between the high- and low-risk groups in the train cohort. (**C**) Time-dependent ROC curves for predicting 1-year, 3-year, and 5-year OS in the train cohort. (**D**) Univariate Cox regression analysis in the train cohort. (**E**) Multiple Cox regression analysis in the training cohort. (**F**) The distributions of risk scores and survival status in the validation cohort. (**G**) Kaplan–Meier curves for the OS of patients between the high- and low-risk groups in the validation cohort. (**H**) Time-dependent ROC curves for predicting 1-year, 3-year, and 5-year OS in the validation cohort. (**I**) Univariate Cox regression analysis in the validation cohort. (**J**) Multiple Cox regression analysis in the validation cohort.

The dataset from TCGA was randomly divided into two groups in a ratio of 1:1, including a training cohort (n = 196) and a validation cohort (n = 194). The median risk score was used to classify two cohorts as HR and LR, respectively. The KM survival analysis of BC patients verified that the HR group exhibited a shorter OS than the LR group (Fig. 5B,G). The survival situation found that the dead cases had an increased risk score, and the heatmap revealed that the eight lncRNAs constructed in the signature were differentially expressed (Fig. 5A,F). For the training cohort, the AUCs of 1-, 3-, and 5-year OS were 0.740, 0.790, and 0.777 (Fig. 5C), and those of the validation cohort were 0.674, 0.690, and 0.698, respectively (Fig. 5H). The univariate Cox regression implied the association of risk score, age, and stage with OS (Fig. 5D,E). According to the multivariate Cox regression, risk score could independently predict the prognosis in the two cohorts (Fig. 5I,J).

Afterward, we focused on scoring signatures to predict survival in different clinical subgroups. The HR group presented a poorer OS than the LR groups (Fig. S3A–L). Generally, our risk prediction signature could accurately predict patient outcomes.

### Construction of nomogram and PCA

**Figure 6:**
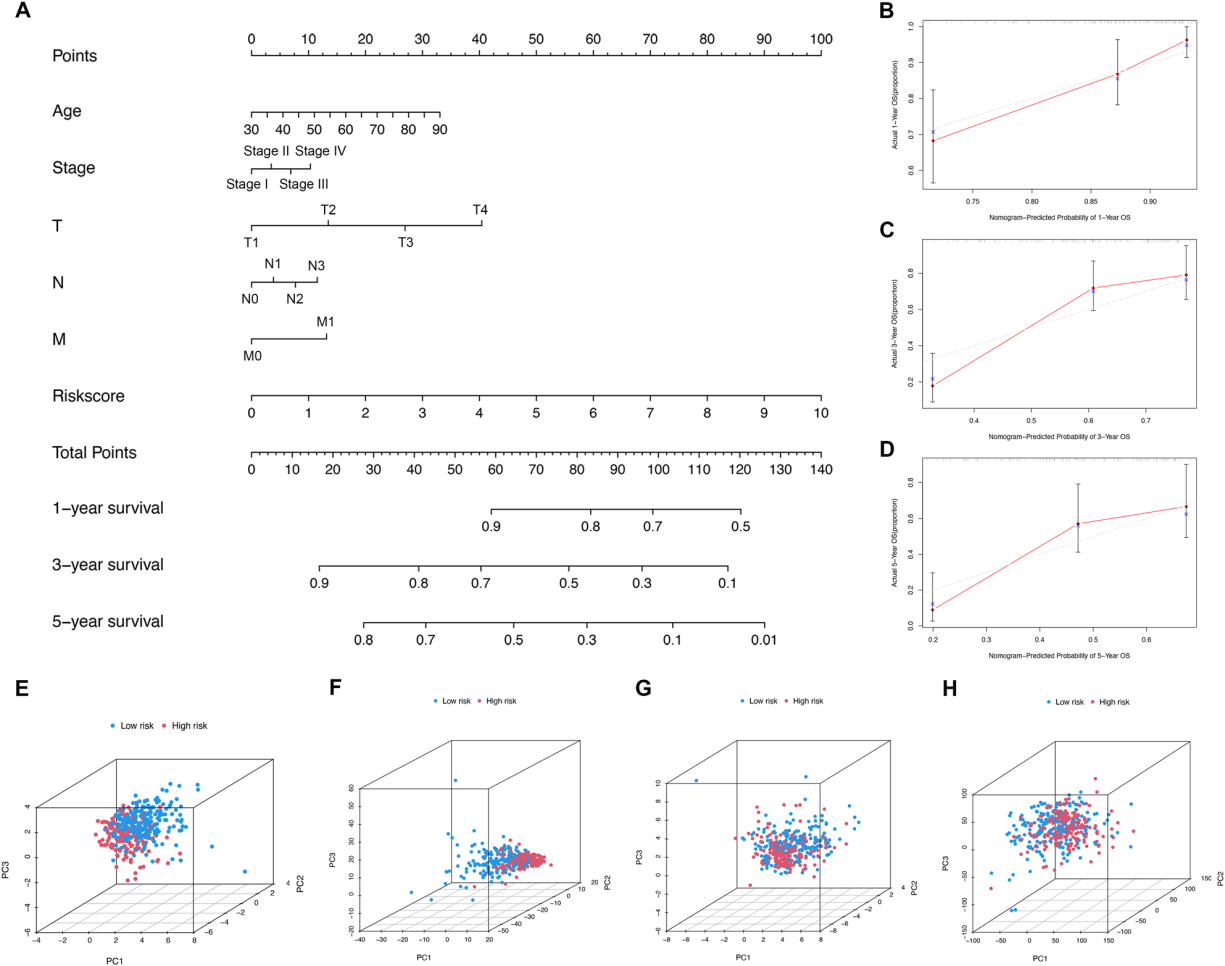
Construction and validation of nomogram to predict the OS of bladder cancer patients. (**A**) Construction of nomogram based on clinicopathological characteristics and risk score. (**B**) The nomogram’s 1-year OS calibration curve. (**C**) The nomogram’s 3-year OS calibration curve. (**D**) The nomogram’s 5-year OS calibration curve. (**E**) Principal component analysis (PCA) of the signature-related lncRNAs. (**F**) PCA of the m7G-related lncRNAs. (**G**) PCA of the m7G-related genes. (**H**) PCA of the all genes.

The clinical factors and risk score were combined to establish a nomogram for the prediction of the 1-, 3-, and 5-year OS to predict the prognosis of each patient with BC (Fig. 6A). Calibration curves indicated consistency between the 1, 3, and 5-year OS estimates from the prediction of the nomogram and the actual overall survival rates (Fig. 6B–D). According to the C-index curve, the risk score had a superior survival prediction than clinicopathological characteristics (Fig. S2C).

In the TCGA dataset, the PCA indicated the distribution of the two risk groups in two directions, indicating that the risk signature-related lncRNAs can more effectively classify BC patients into HR and LR groups (Fig. 6E) than m7G-related lncRNAs (Fig. 6F), m7G-related genes (Fig. 6G) and all genes (Fig. 6H). These data reflected the better identification of the signature-related lncRNAs.

### Gene set enrichment analysis

**Figure 7:**
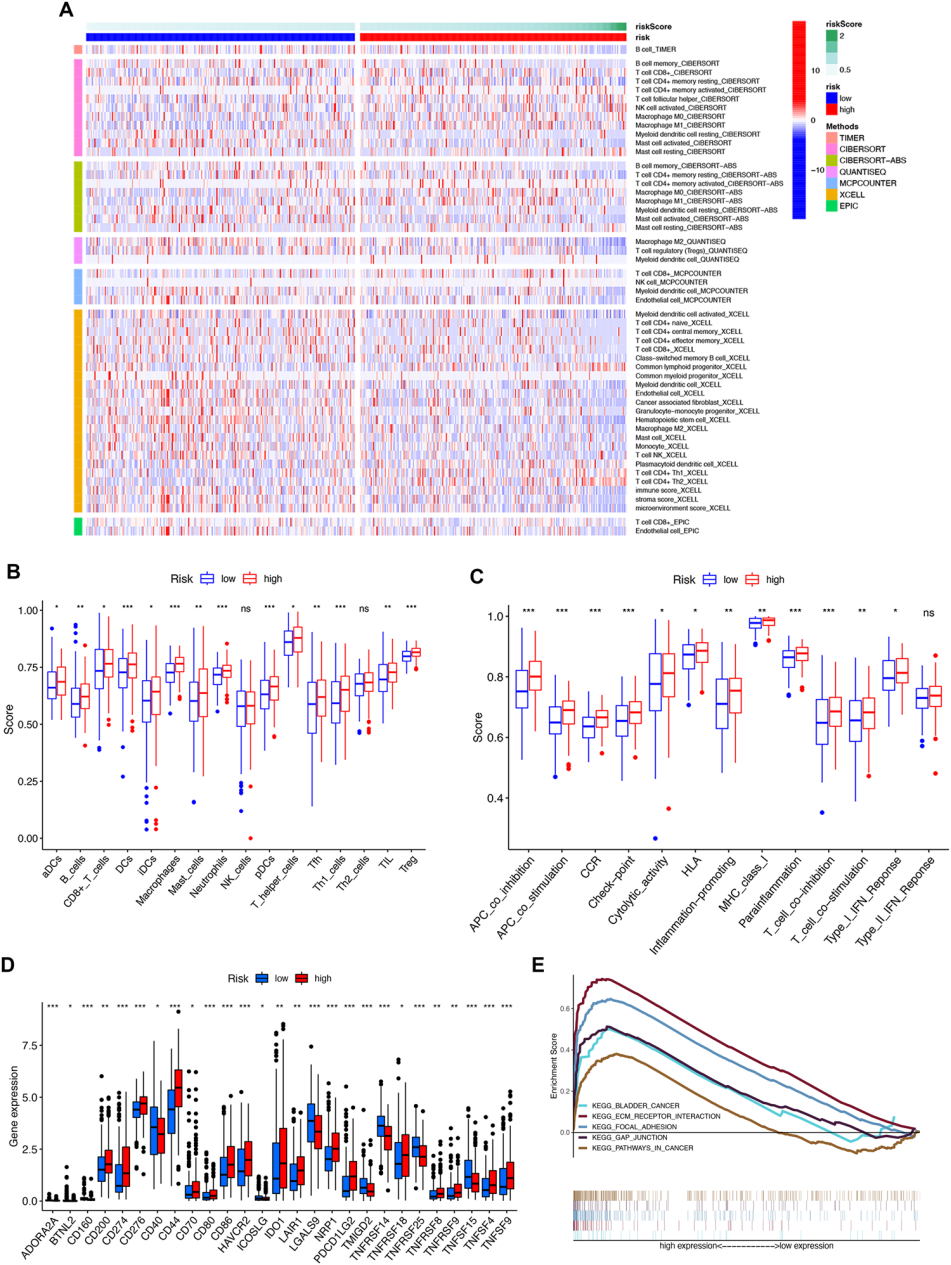
Immune infiltration, immune function, immune checkpoint and gene set enrichment analysis (GSEA) analysis. (**A**) Heatmap for immune cell infiltration landscape based on the TIMER, CIBERSORT, CIBERSORT–ABS, QUANTISEQ, MCPCOUNTER, XCELL and TIMER algorithms among high and low risk group. The heatmap was created by using “pheatmap” package in R 4.2.1 (https:// www.r-project.org/). (**B**) Abundance of 16 infiltrating immune cell types between high- and low-risk groups. (**C**) Abundance of 13 immune-related function score difference in high- and low-risk group (**D**) Expression of immune checkpoints in high- and low-risk groups. (**E**) According to GSEA enrichment analysis, m7G-related lncRNAs were may be involved in ecm receptor interaction, focal adhesion, gap junction, bladder cancer and pathways in cancer. Kyoto Encyclopedia of Genes and Genomes (KEGG) pathway enrichment analysis (Sourced from www.kegg.jp/kegg/kegg1.html). *P < 0.05; **P < 0.01; ***P < 0.001.

The HR and LR groups presented different prognoses. We performed GSEA to investigate the signature-related biological pathways. The results revealed the primary enrichment of the HR group in “ECM-receptor interaction”, “Focal adhesion”, “Gap junction”, “Pathways in cancer”, and “Bladder cancer” (Fig. 7E).

### Analysis of immune cell infiltration, function, and expression of immune checkpoints molecules

The distribution of immune cells in the HR and LR groups identified by the markers was scrutinized by TIMER, CIBERSORT, CIBERSORT–ABS, QUANTISEQ, MCPCOUNTER, XCELL, and TIMER algorithms (Fig. 7A, Table S2). We assessed cumulative scores for various immune cell subsets, associated functions, or pathways by ssGSEA to explore further the relationship between risk score and immune status. The LR group had significantly lower infiltration levels (including CD8 + T cells, macrophages, and Treg) (Fig. 7B). In contrast, the HR group had an abundance of functional immune pathways, including the co-inhibition and co-stimulation of antigen-presenting cells (APCs) and T cells, chemokine receptor (CCR), and cytolytic activity (Fig. 7C). In addition, several immune checkpoint molecules were expressed higher in HR group than LR group, such as CD274, CD276, and IDO1 (Fig. 7D).

### Tumor mutational burden analysis

**Figure 8:**
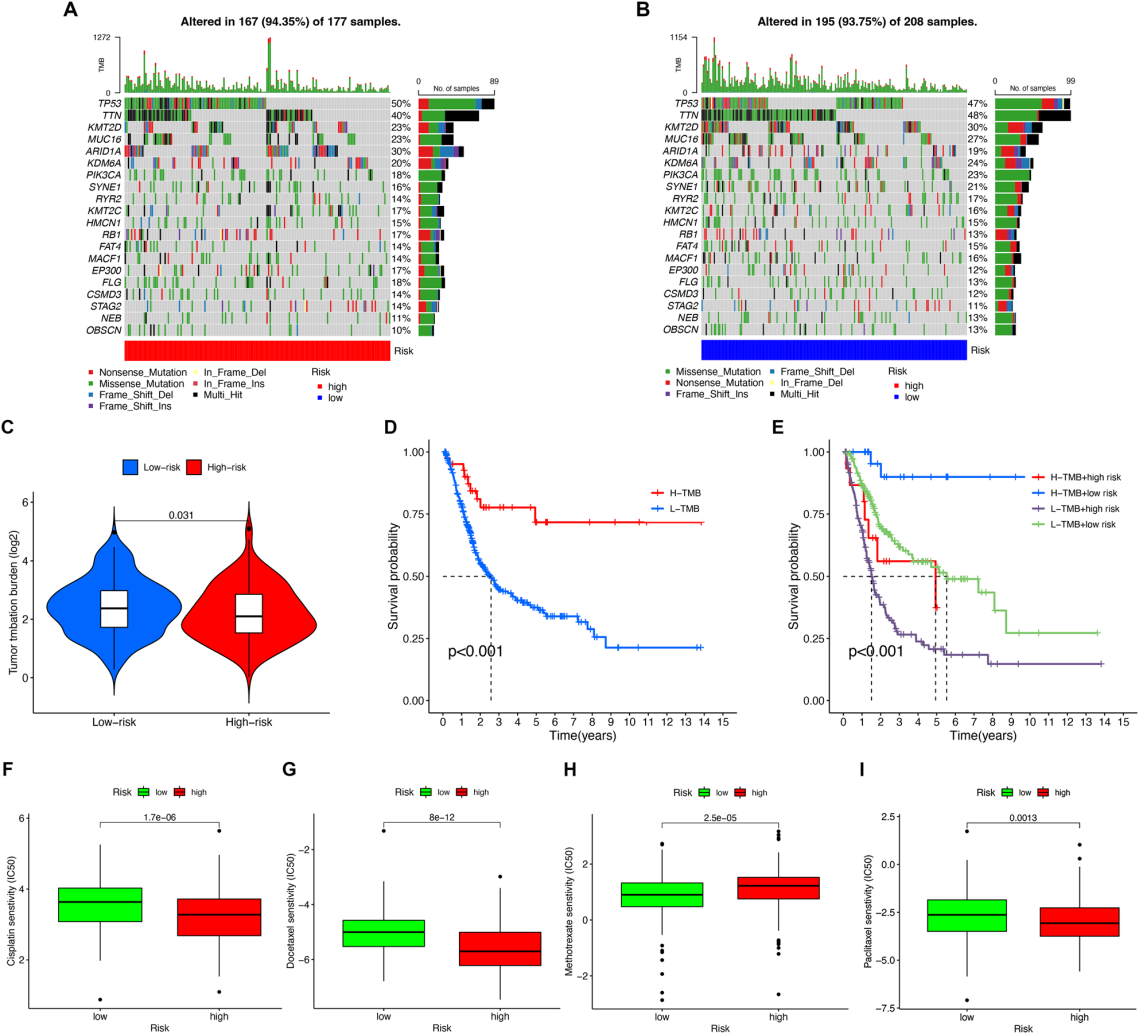
Tumour mutational burden (TMB) and Drug susceptibility analysis. (**A**) The waterfall chart of the frequently mutated genes in the high-risk group. (**B**) The waterfall chart of the frequently mutated genes in the low-risk group. (**C**) The relation of TMB and different risk scores. (**D**) Kaplan–Meier curves of high- and low-TMB on OS. (**E**) Kaplan–Meier curves of TMB and risk score on OS. (**F**–**I**) Treatment effects of cisplatin, docetaxel, methotrexate and paclitaxel were evaluated in patients with high- and low-risk groups.

By calculating the tumor mutation burden, the LR group presented a higher mutation rate than the HR group (Fig. 8C). And the mutation rate decreased with increasing risk score (Fig. 8C). In the HR group, TP53, TTN, ARID1A, KMT2D, and MUC16, with mutation rates exceeding 20%, had the highest mutation rates (Fig. 8A). In the LR group, TTN, TP53, KMT2D, MUC16, and KDM6A had the highest mutation rates (Fig. 8B). Patients who had low TMB presented a shorter OS relative to high TMB (Fig. 8D). Moreover, considering the risk score and TMB together, it was found that patients with low TMB and high risk had the worst prognosis (Fig. 8E).

### Identification of correlation between risk score and chemotherapy drugs

The IC50 was used to assess the therapeutic response of several chemotherapeutic agents. Methotrexate showed higher IC50 values in the HR group (Fig. 8H), and there was greater sensitivity to cisplatin, docetaxel, and paclitaxel in the HR group (Fig. 8F,G,I). Chemotherapeutic drug sensitivity can be assessed using the risk signature.

### Expression validation of lncRNAs in BC Cells

**Figure 9:**
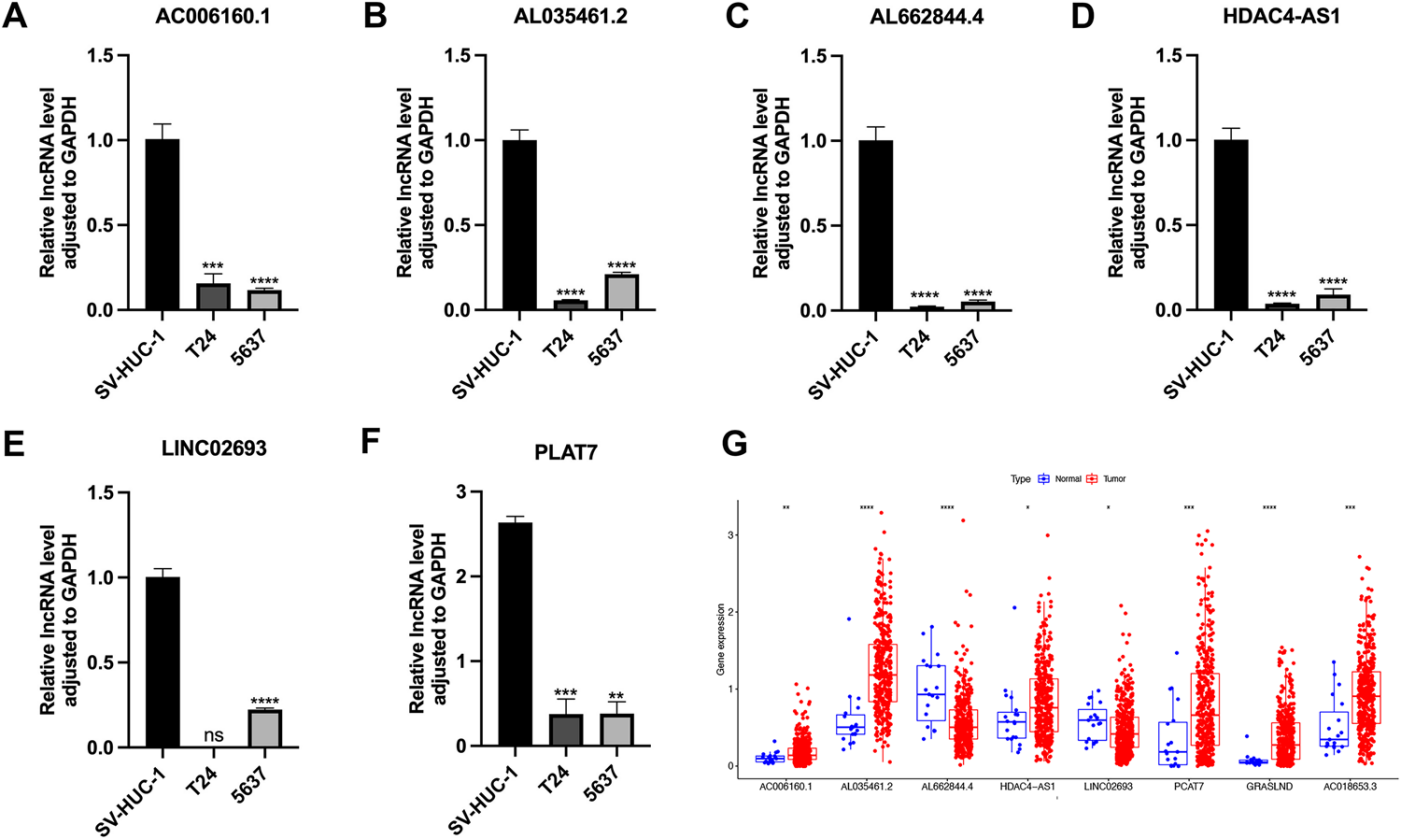
Relative expression of lncRNAs. (**A**) AC006160.1 (**B**) AL035461.2 (**C**) AL662844.4 (**D**) HDAC4-AS1 (**E**) LINCO2693 (**F**) PLAT7 expression in normal (SV-HUC-1) and BC cell lines. (**G**) Heatmap of lncRNAs expression in TCGA. *P < 0.05; **P < 0.01; ***P < 0.001; ****P < 0.0001.

QRT-PCR served for assessing model-associated lncRNAs' expression levels in BC cell lines. Relative to normal cells, AC006160.1, AL035461.2, AL662844.4, HDAC4-AS1, LINC02693 and PCAT7 were decreased in BC cell lines (Fig. 9A–F). There was no statistical difference in the expression of GRASLND and AC018653.3 (Fig. S2D). The expression trends of AL662844.4 and LINC02693 were consistent with the predicted results (Fig. 9G).

## Discussion

There are about 550,000 new BC patients and about 200,000 deaths globally annually^41^. NMIBC is prone to progress to MIBC, 50% of MIBC will metastasize, and patients with advanced metastatic disease have a poor prognosis. Compared with other tumors, NIMBC's frequent relapses and subsequent treatment impose an additional financial burden on patients^5^. Therefore, if a high-accuracy individualized predictive signature for judging prognosis can be implemented, it is possible to make better clinical decisions and thus improve patient outcomes.

We first applied univariate Cox regression to identify 882 lncRNAs related to m7G and then filtered 130 lncRNAs with prognostic relevance. BC patients were then classified into two clusters (1 and 2) by consensus clustering, and the cluster was correlated with the prognosis and clinical stage of patients. Further analysis found that cluster 1 differed from cluster 2 in immunological scores, immune cell levels, and checkpoint analysis. Based on 130 prognosis-related lncRNAs, eight lncRNAs were identified utilizing LASSO and multivariate Cox regression analysis to establish the m7G-related lncRNAs' predictive signature. The risk score cut-off values were considered to divide patients into the HR and LR groups. Survival analyses and risk curves validated the risk score as a clinical prognosticator. The AUCs manifested a gradual upward trend from the first to the fifth year, and the prediction signature had reliable long-term prognostic value. Univariate and multivariate Cox regression further validated that predictive signature could robustly and independently predict BC patients’ OS. Furthermore, the risk score and clinicopathological characteristics served for the development of a nomogram, which is expected to provide patients with a reliable prognostic tool based on m7G-related lncRNAs.

As a biomarker for BC diagnosis and treatment prediction, lncRNA has recently gained attention^42^. Clinical markers could provide essential reference information for the diagnosis and prognosis of tumor patients^43,44^. The roles of more and more relevant lncRNA signatures in BC have been explored through bioinformatics methods. Cao et al.^45^ established an immune-related signature and predicted immunotherapy response in BC patients. A ferroptosis-related signature can be an independent prognostic factor for BC and guide clinical treatment^46^. A signature constructed from m6A-related lncRNAs can identify patients with poor prognosis and suboptimal immunotherapy, thus providing a new approach for BC treatment response prediction and patient stratification^47^. However, the study on m7G-related lncRNAs in BC is insufficient. Therefore, we established a new signature using eight m7G-related lncRNAs that provided prognostic information and tumor immunity. AC006160.1 overexpression can inhibit the proliferation and migration of BC cells^48^. PCAT7 is crucial in prostate cancer bone metastasis, which is upregulated by the activation of TGF-β/SMAD signaling and β signaling, forming a positive feedback pathway^49^. These lncRNAs may affect the initiation and progression of BC through m7G regulation, and the mechanism deserves further exploration.

Recently, there has been renewed interest in immune components due to their application in immunotherapy and their prognostic and therapeutic potential. The immune system plays an important role in the development of cancers and immunotherapy^50^. Notably, metabolic molecules greatly influence the immune environment, thus, the disease progression^51,52^. Besides, recent studies revealed that exosomes carried molecules (including lncRNAs), which showed high antitumor activity in a variety of tumors, promote the expansion of regulatory T cells, inhibit the proliferation and activation of CD8 + T cells, and play an immunosuppressive role^53^. Our study showed that the HR group had more active immune cells than the LR group. CD8 + T cells are important in suppressing tumor growth. However, a study revealed a positive correlation between the abundance of invasive T cells with epithelial-mesenchymal transformation (EMT), with high T cells in tumors located in the stroma without interacting directly with cancer cells, rendering these T cells ineffective as antitumor drugs^54^. Therefore, we speculated that m7G-related lncRNAs might be correlated closely with EMT, resulting in poor patient prognosis. Regulatory T cells are core factors in mediating immune tolerance and can use multiple mechanisms to mediate inhibition, thereby silencing anti-tumor immune surveillance or preventing tissue damage by activated T cells. Differences in immune checkpoint expression indicated that LR group patients had lower levels of immune checkpoint infiltration, which promoted immune tolerance and affected patient prognosis. An inverse relationship exists between risk score and TMB, which markers the immune checkpoint blockade (ICB) biologically. A recent study has shown that patients with high mutational burden BC have favorable response rates to ICB therapy^55^. On this basis, we believe that immunosuppression possible leads to the weak prognosis of the HR group, and the m7G-related lncRNA signature can assess the immune microenvironment and the response to immunotherapy.

In the GSEA analysis, the HR group was associated with ECM receptor interaction, focal adhesion, gap junction, BC, and pathways in cancer. Studies have shown that modulating focal adhesion can enhance the interaction between tumor cells and the extracellular matrix (ECM) and activate signaling pathways that promote tumor growth and metastasis, including invasion, EMT, tumor angiogenesis, and stromal fibrosis^56–60^. The gap junctional intercellular communications act as a positive regulator of EMT and promote tumor metastasis^61^. Among the most frequently mutated malignancies, BC is third only to lung and skin cancer in terms of mutation frequency^62,63^ and has a higher TMB than most other cancers^64,65^. Our study found that the most frequently mutated genes included TP53, TTN, KMT2D, and MUC16 in both groups. Mutant TP53 accumulates in tumors, which promotes tumor cell proliferation, migration, and invasion and enhances drug resistance^66^. These findings facilitate further studies on their role in BC.

However, this study has some shortcomings. First, the signature of m7G-related lncRNA is only constructed and validated in the TCGA database, and external databases are required to validate the performance of the signature. Secondly, due to differences in cell lines and limitations of the TCGA database, the sample size of the normal group was small, which may be the reason for the inconsistency between the qPCR verification results and the predicted results. Next, because of differences in cell lines or extremely low expression, GRASLND was not detected in tumor cells, and AC018653.3 was not detected in normal bladder epithelial cells and tumor cells, so it is necessary to include a larger sample size for relevant experimental analysis. Finally, the regulatory mechanism of m7G-related lncRNAs has not been fully elucidated, and relevant basic experiments are needed to explore further.

## Conclusion

To summarize, a novel predictive signature was constructed according to m7G-related lncRNAs with robust and independent prognostic power to predict patients' responses to immunotherapy. Our analysis initially explored the significance of m7G-related lncRNAs in BC and provided a theoretical basis for m7G-related lncRNAs as the underlying therapeutic target in BC patients.

## Supplementary Information

Supplementary Figure S1.

Supplementary Figure S2.

Supplementary Figure S3.

Supplementary Table S1.

Supplementary Table S2.

## Data Availability

The datasets generated and analysed during the current study are available in the TCGA repository (http://portal.gdc.cancer.gov/).
