# Supplementary material for: An m7G-related lncRNA signature predicts prognosis and reveals the immune microenvironment in bladder cancer
Source: Sci Rep. 2023 Mar 15;13:4302. doi: 10.1038/s41598-023-31424-y (PMC10017825; doi:10.1038/s41598-023-31424-y)
Supplement: Supplementary file 4 — Supplementary Table S1. [file 41598_2023_31424_MOESM4_ESM.docx]

**Supplementary Table S1. The primer sequences of m7G-related lncRNAs**

| Symbol | Sequence (5’-3’) |
| --- | --- |
| AL662844.4-QPCR-F | TGGTCTACGGAAAATGACAAGT |
| AL662844.4-QPCR-R | GTCTGCATTTTCACAGCGG |
| HDAC4-AS1-QPCR-F | GCATGGCTGCCTAGCGGAGT |
| HDAC4-AS1-QPCR-R | TGTCAAAGGCGCTTCCTCCC |
| LINC02693-QPCR-F | AGACGAGCAAAGCAGCAGAA |
| LINC02693-QPCR-R | GGCAGGGGAGATGATTCTTGG |
| AC006160.1-QPCR-F | ATGCCTGGAGAGACTTTGGC |
| AC006160.1-QPCR-R | GCCTGTCTTGTTCCCGCTAT |
| AC018653.3-QPCR-F | AGAGACGCACCTCTGCTACT |
| AC018653.3-QPCR-R | GCCAGACCTCCTGAGAACAT |
| AL035461.2-QPCR-F | ATATGCCTGTGGTTCCAGCTAC |
| AL035461.2-QPCR-R | ACATTGCACTCCACATAGACTCG |
| GRASLND-QPCR-F | TCCTGAGACCTGCCTACGAA |
| GRASLND-QPCR-R | GTGTGTTCCCTCGGTGTAGG |
| PCAT7-QPCR-F | AACAAGCCAACCGCACAATC |
| PCAT7-QPCR-R | CATGGGTTGTGGAGAGGGAC |
| GAPDH-QPCR-F | TCAAGAAGGTGGTGAAGCAGG |
| GAPDH-QPCR-R | TCAAAGGTGGAGGAGTGGGT |
